# On the Connexion between Morbid Physical and *Moral* Phenomena

**Published:** 1856-10-01

**Authors:** J. F. Denham


### Art. V.—ON THE CONNEXION BETWEEN MORBID PHYSICAL AND *MORAL* PHENOMENA.

BY THE REV. J. F. DENHAM, M.A., F.R.S.

[No. VII. of a Series.]

### Appendix I.

THE general proposition about to be maintained is, that deviations from moral rectitude in the thoughts, dispositions, and actions of mankind are associated with bodily disease, and caused by it; understanding, however, by the latter term literally *all* states and conditions of the body removed from its normal condition—beginning with the state of simple bodily excitement produced by evil ideas addressed from without to the mind and affecting the body through the mind; and including the morbid effects on the mind of physical excitement applied to the body; and all the morbid alterations invariably produced on the mind and moral powers by functional or organic diseases, malformation, &c. We are about to adduce reasons for the opinion that an abnormal or morbid condition of the body of some or other kind or degree, is the *proximate* cause of vice, sin, and crime ; and that since such a condition of the body and its morbid effects on the mind constitute *insanity*, either latent or active, partial or entire, temporary or chronic, that vice, sin, and crime originate in insanity in the *comprehensive* and philosophical sense of that term. It will be allowed, on all hands, that vice, sin, and crime are *effects;* and we are about to investigate the *proximate causes* of those effects. We wish to find an intelligible and probable answer to the questions—Why any human being becomes vicious, sinful, or criminal ?—Why all human beings are not similarly and equally vicious, sinful, and criminal ?—and Why any human being is not at all times similarly and equally vicious, sinful, and criminal ? And we believe that the answer to these questions is to be found in the differences of the physical condition of different individuals, and in the difference of the physical condition of the same individual at different times with reference to health and disease, however produced.

In behalf of this opinion an appeal will first be made to our consciousness and experience ; secondly, to our observation ; thirdly, to the authority of the Scriptures ; fourthly, to the opinions of the Catholic fathers ; fifthly, to the Articles and other documents of the Church of England ; sixthly, to the teachings of its standard divines ; seventhly, to the involuntary concessions of the adversaries to this opinion. We shall, lastly, offer some practical conclusions from our subject. First : We appeal to the consciousness and experience of every individual, and we ask, in regard of those deviations from moral propriety with which all considerate persons will, we believe, acknowledge themselves to be too frequently chargeable—whether they are not conscious, upon due attention to their mental and moral states, that a suggestion, temptation, inducement, occasion, or motive to any of these deviations of any kind and degree whatever, does not then, only, become effectual when after that a certain kind of physical *excitement* has been sensibly produced in the brain circulation, &c.—whether they are not conscious that some or other portion of our physical nature must first become *involved and coactive* with the suggestion, temptation, inducement, occasion, or motive,—and whether it is not, through means of such a physical coaction, continued and increased, that the balance between the moral powers and inclination is ultimately destroyed? We appeal to the experience of all persons whether their only safety from an evil suggestion, &c., does not lie in the resolute suppression of it before such a physical coaction has been effected—whether, if they have often neglected such a suppression, the physical coaction does not become more readily effected, and whether, in the case of a continued neglect of such a suppression, the physical coaction and the compliance with the temptation, &c., do not become irresistible? We appeal to their experience of themselves in regard of what is commonly called their "infirmity," "failing," "weak point," or "besetting sin," or what they are "given to"—whether we have not now set before them the true account of its origin and power over them. What, indeed, is meant by the common phrase "keeping the mind calm," but the prevention of a physical perturbation? It is possible that a previous morbid state of the body or physical defect may render the prevalence of the temptation more easy; but even in this case the coaction with it of the physical portion of our nature is clearly perceptible. We further appeal to the experience of every man whether he has not often found that a diseased or disordered state of his body has been attended with an inclination or tendency to some or other kind of immorality, either of thought, disposition, or action,—and whether, in proportion as his bodily state approaches the condition of health, such an inclination or tendency is not diminished. Is any man equally inclined to be amiable and virtuous under all the different states of his bodily health and strength,—under a careful moderation and selection in regard of his food, a due attention to exercise, &c.—and under a system of excess, negligence, or indolence? We believe that the reply to these appeals will afford a decided presumption in favour of our opinion respecting the physical origin, or at least proximate cause, of vice, sin, and crime; for since all persons are conscious of such an origin and proximate cause in regard of minor immoralities, it is, we think, only fair and safe to infer a similar origin and proximate cause in regard of those greater immoralities of which mankind are guilty; agreeably to Sir Isaac Newton's second rule of philosophising, that "phenomena of the same sort are to be accounted for by the same cause."

Secondly, we appeal to the observation which all persons are more or less constrained to make on their fellow men. Let, then, any person direct his view to his acquaintances and friends, and does not the certainty of hereditary vices, sins, and crimes, and of vices as associated with particular bodily constitutions, conformations, states and conditions, present itself with irresistible conviction to his mind? Who has not observed instances among mankind of what Shakspeare calls—

> "Some vicious mode of nature in them,
> As, in their birth, (wherein they are not guilty,
> Since nature cannot choose its origin,)

By the o'ergrowth of some complexion,
Oft breaking down the pales and forts of reason;
The stamp of one defect
Being nature's livery, or fortune's star."*

Who has not had reason to say—

"Blessed are those
Whose blood and judgment are so well commingled,
That they are not a pipe for Fortune's finger
To sound what stop she please:
That are not passion's slaves?"†

Perhaps the most palpable instance of the connexion between morbid physical and moral phenomena is afforded by intoxication and its consequences on the disposition and conduct. In Dr. Lettsome's well-known scale of Drunkenness, the vices, sins, and crimes vary in degrees of atrocity proportionably to the strength of the different alcoholic stimulants,—that is, according to the degrees to which a morbid state of the physical portion of the drunkard's system is produced. Even constitutional exciteableness alone may, if indulged, lead to many immoralities. Hysteria in children of both sexes is often attended with a propensity to falsehood, hypocrisy, and other forms of vice. So is also a precocious or rapid development of the juvenile constitution, whether male or female: such cases either terminate in the subsidence of immoral propensities along with the completion of the physical development, or in death. I find in my memoranda the case of a female child of about nine years of age, of premature growth, excited manners, peculiarly sparkling eyes, keen-minded and mischievous, who would, unless closely watched, approach visitors to the house in a most winning manner, and, having gained their attention, would violently thrust her finger into their eyes; and having accomplished her purpose, would burst out into triumphant laughter. She died before she was ten years old, of fever. It is, I believe, universally admitted that insanity of all kinds is attended with a propensity to some or other kind and degree of immorality, as is also mental weakness or a deficiency of understanding.

I give the following instances that have come under my notice: they may be considered as types of classes containing many individual cases:

A young man, aged 23, of delicate constitution, defective moral and religious education, habitually talkative, boastful, prone to exaggerate, but yet possessing a remarkable aptitude for drawing, was, during the last few years of his life, addicted to sensuality and the company of the dissolute; seemed totally

* "Hamlet," Act 1, scene iv. † Act 3, scene ii.

destitute of all ideas of justice to God, to himself, or his fellow-creatures. Even a short time before his death, and when he was supposed to be too weak to need surveillance, he managed to rise from his bed, and was found at the distance of several miles from his abode, in a most deplorable state, in one of the lowest haunts of profligacy. The cause of his death, according to the opinion of his medical attendants, was diseased heart combined with phthisis. Often have I seen a family plunged into distress by the early and sometimes sudden indications in some one of the younger members of it of waywardness, proneness to immorality, insensibility to all expostulations and reasoning, and even to any consideration of self-interest. The result has been, generally, insanity or early death. "Enemies to themselves" is the popular comment in such instances; and I have had reason to believe that there was in some of these cases a real hatred of themselves, a wish to degrade and ruin themselves, and an impatience till they had thoroughly succeeded.

CASE 2.—A young man, of delicate appearance and very excitable temperament, thin, pale, and diminutive for his age, introduced himself to me as the son of a clergyman, and complained of an incessant inclination to abandon himself to immorality, although, as he assured me, he was a perfect stranger to the vice to which he was tempted. It had, however, become his fixed idea, although he responded, and apparently with sincerity, to my exhortations and warnings. I heard a few months afterwards that he died in an asylum for the insane.

CASE 4.—Reported to me by the survivor. A young man, remarkable for the correctness of his demeanour, was many years ago proceeding one evening to chapel at Cambridge, accompanied by a fellow-student and friend, when he suddenly proposed to abandon chapel and resort to the haunts of dissipation. His friend expressed his astonishment at this unexpected development in his character. He nevertheless turned away, was absent all night from his college, and three weeks afterwards he died raving mad.

CASE 5.—A young female, who had always evinced some degree of mental and bodily weakness, vanity, singularity, proneness to squander money, was attacked, at about seventeen years of age, with mental aberration, accompanied with well-defined catalepsy, attended with præternatural acuteness of the senses. She appeared able to discern by her smell whatever was brought into the house, though confined to the highest room in it. She knew who came into the house, and heard their footsteps when too distant for others to hear them; was sullen and violent, and unless prevented, would feel after those who came to see her and bite them, and expressed her disappointment by rage and hideous noises. One medical attendant pronounced her "a demoniac, such as we read of in the time of Christ;" another, more wisely, predicted recovery upon a change in constitution. His prediction was fulfilled, and for some years after she filled an humble station of usefulness.

Another case was of a female of nearly the same age, of plethoric constitution, who, after a severe fright, evinced symptoms of malignity and frenzy, and the same disposition to bite persons. In order to effect her purpose, she feigned sanity in the most plausible manner, bit the person who believed her, and expressed her satisfaction at having done so with derisive laughter.

Another young female also showed a strong propensity to mischief and falsehood, along with decided indications of insanity. She accused the servants of theft, and secreted articles herself, in order to bring their honesty into suspicion. She nevertheless expressed a strong desire to be confirmed; could not be made to understand her moral unfitness for the rite, and ultimately, by an ingenious stratagem and deception, managed to receive it at a distance from her own neighbourhood. I have known instances of young females who, along with similar mental and moral characteristics, evinced an impetuous, unintelligent wish to change their religion, be baptized by immersion, take the veil, &c.

A female in a workhouse, considered insane by all around her, repelled all religious instruction, would not be present at prayers if she could avoid it, and repeatedly replied with great apparent animosity to all my exhortations to confidence in God, "He did not save my brother," whose death by the hands of justice had, I was informed, caused the overthrow of her reason.

I have met with several instances in which a severe illness or accident had greatly altered the moral dispositions. A man received a blow on his head by being thrown out of his gig, and to the end of his life was remarkable for pride, which had not been particularly observed in him previously. A young man, after a fit of epilepsy, became habitually conceited, and manifested a strong propensity to immorality. Instances of the association with diseased heart of horrid thoughts, temptations to vice, and especially to inebriety, have often come under my notice. Frequently have the sufferers complained that they "felt everything affect their heart," and have stated that a sensible perturbation of that organ preceded their dreadful ideas, inclinations to violence and crime, dejection and despair, thoughts of self-destruction, &c. None but those who have ministered to such sufferers can form an idea of the extent and variety of the abnormal ideas, feelings, and inclinations by which they are frequently assailed.

Thirdly. We now proceed to adduce the references contained in the Scriptures to the association of bodily disease, in the comprehensive sense of the term already assigned to it, with vice, sin, and crime, and according as these references occur in a chronological arrangement of the Old Testament, Apocrypha, and New Testament. I request permission, however, to reproduce here, from No. 3 of these papers, the leading assertions of the Scriptures respecting the degenerate and damaged state of the body or "flesh," as they frequently term it, and respecting the consequences of various kinds produced on the mind, soul, or spirit, by its union with these intellectual and emotional principles of our nature. It was there shown that the Scriptures acquaint us with an immense deterioration that was inflicted on the body and external circumstances of the first parents of the human race, in consequence of their transgression, and entailed upon all their posterity; that this deterioration is assigned by the Scriptures themselves as the proximate cause of the existing phenomena of the perturbed state of the mental and moral nature of man, and that extraordinary instances of these phenomena are ascribed to those additional physical disturbances by which the state of the body is well known to be still further removed from its normal condition; that St. Paul declares that "the flesh lusteth against the spirit, and the spirit against the flesh; that they are contrary the one to the other, so that we cannot fully do the things that we would;" that he thus describes his own state: "In me, that is, in my *flesh*, dwelleth no good thing. I find a *law* that when I would do good, evil is present with me. I delight in the law of God after the inward man, but I see another law in my *members warring against the law of my mind*, and bringing me into captivity to the law of sin which is in my members. So then with my *mind* I myself serve the law of God, but with the *flesh* the law of sin;" that he asserts the "disposition of the flesh is enmity against God; it is not subject to the law of God, neither indeed *can* be;" that both St. Paul and the other Apostles perpetually use the terms "sinful flesh," the "infirmity of the flesh," "weak through the flesh," the "lusts of the flesh," our "vile" "mortal bodies;" and exhort their readers to avoid "the lusts of the flesh which war against the soul," and "to mortify the deeds of the body," as the indispensable means of virtue and salvation; that St. James gives the following account of the origin and progress of sin: "every man is (effectually) tempted when he is drawn away of his own lust and enticed: then when lust hath conceived, it bringeth forth sin;" that St. Paul includes among "the works of the flesh" not only the more obvious sensualities, but even "idolatry, witchcraft or poisonings, hatred, variance, emulations, wrath, strife, seditions, heresies, envyings, murders," and declares that "he keeps under his body, and brings it into subjection, lest by any means when he had preached to others he himself should be a castaway."

I now beg to add, that, according to the Hebrew psychology, which has every likelihood of being the true and correct, the body and mind of man are represented as constituting one entity, so that whatever affects the body may be reasonably expected to affect the mind, and *vice versâ*. I beg to state this biblical principle of the unity of man in the language of Milton, who, of all students of Scripture may be expected to be fully acquainted with its theory of human nature.

"Man having been created after this manner, it is said, as a consequence, that 'man became a living soul,' whence, it may be inferred —unless we had rather take the heathen writers for our teachers respecting the nature of the soul—that man is a living being, intrinsically and properly *one*, and individual, *not* compound, or separable; not, according to the common opinion, made up and framed of two distinct and different natures, as of soul and body, but that the whole man is soul, and the soul man—that is to say, a body or substance individual, animated, sensitive, and rational; and that the 'breath of life' was neither a part of the Divine essence nor the soul itself, but, as it were, an inspiration of some divine virtue, fitted for the exercise of life and reason, and infused into the organic body; for man himself, the whole man, when finally created, is called, in express terms, *a living soul*. God having completed His whole work of creation, and rested on the seventh day, it would seem, therefore, that the human soul is not created daily by the immediate act of God, but propagated from father to son in a natural order."

It appears probable that this doctrine of the unity of man was known to some of the more ancient Greek philosophers, and its consequences also recognised by them, viz., that not only whatever affects the body must also necessarily affect the mind, but that the body itself is, according to its several states, the origin of corresponding ideas to the mind.* So also in later times, Gaubius remarks:

"The mind itself and the body, which are things, according to the opinion of most men, extremely different; when they coalesce to constitute man, they associate so intimately, and with so close a contexture, that they seem to penetrate each other; and if we may use the chemist's phrase, we may affirm, that they melt one another down into

* "Lewes's Biographical History of Philosophy," vol. i. p. 89, &c.

one common mass, so that whilst life remains in vigour, where the mind is, there the body is, and wherever the body is, there the mind is also; nor can there scarce a particle of us be found in which a mixture of each may not be discovered."*

"The mind perceives differently according to the various conditions of the body to which it is joined, and she may be disturbed by the body in her operations, and at some times be hindered from thinking as she would, and at other times be compelled to *think* according as the body commands."

More towards our own times Dr. Feuchtersleben remarks:

"Matter and spirit, when they are united to form body and mind, can no longer be considered otherwise than as *unity*. No one who is acquainted with human nature will deny that those peculiar maladies of the mind, error and vice, originate frequently in states of the body."†

In still later times, Wilkinson, in his work on "The Human Body and its Connexion with Man," has exhausted the application of the unity of human nature and the intellectual and moral coaction of the body with the mind.

But the Scriptures, as is well known, contain in the earliest portion of them, an account of the introduction of moral evil among mankind, and we think that account includes precisely such a coaction of the physical, mental, and moral part of our nature as we have already, by our appeal to our consciousness and experience, shown to attend the perpetration of moral evil to the present hour. We think that, according to St. James, "lust," or bodily desire, excited in the first instance by an evil idea coming from without, and by its consequent disturbance of the physical part of Eve's nature, and by that disturbance overwhelming her moral powers, was the completing cause of the primal transgression. "The serpent"—whatever may be meant by the term—(for we hold with Dr. Jortin, that this question is immaterial, since all temptation succeeds upon the same principles, and is to be repelled by the same means)—"the serpent" first calls the *attention* of the woman to the nature of the prohibition imposed upon herself and her husband, in the following dangerously suggestive language: "Yea, hath God said ye shall not eat of every tree in the garden?" Now the mere act of having the attention called to the nature of an object, causes, as no doubt does every idea, a change, to some extent, in the brain, the nervous system, and the entire physical constitution, so that the status of the subject of such attention is never, phy-

* "A Philosophical Discourse on the Management and Cure of the Disorders of the Mind," by H. D. Gaubius. Translated by J. Taprell, M.D., p. 2.

† "Medical Psychology." Translated for the Sydenham Society, p. 73.

sically, intellectually, or morally, *precisely* the same afterwards as before. We all know, too, the importance that the mind of a child, or of an innocent female, should *never*, even in a single instance, have even its attention called to any idea that may possibly become injurious. Yet up to this point the nature of Eve, though in an altered state, was not so far changed as necessarily to induce the act of transgression. She was, however, in some degree prepared for it, though not irretrievably. But her dangerous attention to it, and the injurious effects upon her of such attention, were still further increased by her *reply*, by her "parleying," as it is called, with the temptation; when she said unto the serpent, "We may eat of the fruit of the trees of the garden, but of the fruit of the tree which is in the midst of the garden, God hath said, Ye shall not eat of it, neither shall ye touch it, lest ye die," which *latter* words being not recorded in the original prohibition, would seem like the result of excitement. However, the very recital of the prohibition would increase her danger, for it would increase her attention to the forbidden object, and to the *restraint on her liberty* connected with it, and her mind would thereby be still further prepared to yield to the bold assertion of the tempter, "Ye shall not surely die, for God doth know that in the day ye eat thereof, then shall your eyes be opened, and ye shall be as Gods, knowing good and evil." Her perilous attention to the entire subject and perception of restricted liberty would now be still further increased, but still the temptation does not succeed until her physical appetite for food becomes also interested in it, for according to the narrator, "and when the woman saw that the tree was good for food, and that it was pleasant," (or, as in the margin, a desire to the eyes,) "and a tree to be desired to make one wise, she took of the fruit thereof and did eat, and gave also unto her husband with her, and he did eat." Such was the process, consisting partly of an evil idea, addressed from without to the mind of Eve, and partly of the commotion in the physical portion of her nature, excited by that idea and increased still further by her own contemplation of it, whereby her moral perceptions were ultimately overwhelmed. We think we see this process recognised by herself in her reply to the question, "What is this that thou hast done?" when she said, "The serpent beguiled me," or "*elated*," or "*puffed me up*," as Bishop Horne translates the word rendered "beguiled" in our version; and St. Paul remarks that the serpent "beguiled" (ἐξηπάτησεν) Eve by his subtlety. Who can believe that during the progress of the temptation to its conclusion, that the circulation of her blood was as entirely calm as it was before the evil idea was presented to her attention—that her cheek did not glow with unwonted ardour—that her quicker breath did not fan her lips, disparted with wonder, curiosity, and ambition? Who can doubt whether the final act was not the result of that irresistible impulse which, to this hour, is the *immediate* cause of compliance with any temptation that the mind has contemplated, until an impetuous tumult of excited feelings has been produced? We think that the nature of intoxication and its effects on the mind and moral powers afford a strong resemblance to the several phases of this as well as of every subsequent premeditated transgression. Accordingly, Milton, with no less philosophical than scriptural accuracy, represents that,

> "In her cheek *distemper flushing glow'd*, [that she was]
> Heighten'd as if with wine, jocund and boon."

Nor does he omit to mention "the fruit" as one "which to behold might tempt alone," or to make "the guileful tempter" descant on the physical attractions of "the goodly tree."

> "Loaden with fruit of fairest colours mixed,
> Ruddy and gold:
> And from whose boughs a savoury odor blown;
> Grateful to appetite, more pleased my sense
> Than smell of sweetest fennel, or the teats
> Of ewe or goat dropping with milk at even,
> Unsuck'd of lamb or kid, that tend their play."

The poet thus particularizes the agency of physical causes,

> "Meanwhile the hour of noon drew on, and waked
> An *eager appetite*, raised by the *smell*
> So savoury of that fruit, which with desire,
> Inclinable now grown to touch or taste,
> Solicited her longing eye."

He thus describes the completion of the act—

> "What hinders then
> To reach, and feed at once both body and mind?
> So saying, her rash hand in evil hour
> Forth reaching to the fruit, she pluck'd, she ate;
> Greedily she engorged without restraint,
> And knew not eating death."*

Although, however, temptation of every kind proceeds and prevails by means of raising a similar disturbance in the physical part of our nature, yet moral evil achieves an easier conquest over fallen humanity, in consequence of "the weakness of the flesh," and "the law of sin" inherent in its members. It seems also probable, that every good or evil action, or even idea, serves to substantiate a corresponding structure in some or other part of the body that facilitates the repetition of such an action

* "Paradise Lost," book 9.

or idea, and that such a structure constitutes the principle of good or evil habits. It is also certain, that when once the moral principle is overwhelmed and the dominion of vicious habits is established, and especially when in such a case, our nature is subjected to physical or mental excitement, the progress of depravity may resemble that "of a bowl down a hill, that increases its motion by going, and will not be stopped or diverted," and that the enormities resulting from moral insanity may be boundless.

In pursuing our Scriptural investigations, we find the murder of Abel by his brother Cain ascribed by the writers of Scripture to "hatred;" but both hatred and murder are by St. Paul enumerated among "the works of the flesh," as also that "wrath" and those other evil passions indicated by Cain's "fallen countenance." Moses also intimates the connexion between polygamy and depravity, and evidently dates from the marriage of the sons of God (or his worshippers) with all the daughters of men (or the irreligious), whom they chose, "the great wickedness that was in the earth" before the Deluge. The most learned commentators also point out physical and *moral* as well as religious reasons for the prohibition of "eating blood" imposed upon the family of Noah and his descendants; and also for many of the Mosaic regulations respecting food. In the Book of Job, which comes next in our chronological order, that patriarch evinces his acquaintance with the tendency of feasting to produce impiety, and fears lest his "sons," during their several days' festival, may have "cursed" or forsaken "God in their hearts." Satan suggests that "if God would put forth his hand and touch or afflict Job's bone and his flesh, Job would *curse* Him to his face." Satan received permission to make the trial, and Job, in the anguish of his bodily pain, "cursed his day," and said many things, both then and afterwards, that fully illustrate the maddening effects of intense bodily suffering on the mind, &c. Eliphaz argues: "What is man that is born of a woman that he should be righteous?" Bildad asks, "How can he be clean that is born of a woman?" where the physical origin of man seems admitted as the invincible cause of his moral imperfection. Job thus expostulates with the Almighty as a reason for forbearance: "Hast thou eyes of *flesh?* or seest thou as man seeth?" In this early book we find the important concession that moral evil is sometimes associated with a deficiency of mental strength, or of natural understanding. "Wrath killeth the foolish man, and envy or indignation slayeth the silly one." Here, too, we find one of those numerous and diversified references to the *heart* which abound in the Scriptures. Job complains, "God maketh my *heart soft,* and the Almighty *troubleth* me:" which seems like an allusion to the timidity connected with such a physical state of the organ. It is frequently considered that such references as this, and those others that will be subsequently selected out of a multitude, are to be regarded as metaphorical expressions. But we think that the remark of Sherlock applies to them that "metaphors do not arise out of nothing." May we not rather suppose that such expressions are derived from actual observations made by priests, sacrificers, embalmers, and diviners?

Returning to the historical books of Scripture, and omitting the instances quoted in the previous paper, we find the immoral effects of the excess of wine remarkably illustrated in the case of the two priests Nadab and Abihu, who together with all the people, had experienced terror at the manifestation of the Divine presence, yet shortly afterwards attempted to celebrate the divine service in a state of inebriation, and were miraculously punished with instant death. The idolatry of Solomon himself is ascribed to his voluptuousness and old age. The writer of the book Ecclesiasticus also remarks that "He bowed his loins unto women, and by his body he was brought into subjection." Repeated references occur to the debasing effects of sensuality on the moral principles. It is particularly enjoined on "kings and princes not to drink wine or strong drink, lest they drink and forget the law, and pervert the judgment of any of the afflicted." "Whoredom and wine, and new wine, take the heart." The slave of lewdness thus testifies to its tendency to lead to still further depravities, "I was almost in *all evil* in the midst of the congregation and assembly."

(*To be continued.*)

